# Hepatitis B virus reactivation with corticosteroid therapy in patients with adrenal insufficiency

**DOI:** 10.1002/edm2.71

**Published:** 2019-05-09

**Authors:** Masako Hatano, Toshihide Mimura, Akira Shimada, Mitsuhiko Noda, Shigehiro Katayama

**Affiliations:** ^1^ Department of Endocrinology and Diabetes Saitama Medical University Saitama Japan; ^2^ Department of Rheumatology and Applied Immunology, Faculty of Medicine Saitama Medical University Saitama Japan; ^3^ Kawagoe Clinic Saitama Medical University Saitama Japan

**Keywords:** adrenal insufficiency, corticosteroids, hepatitis B virus reactivation, rheumatic disease

## Abstract

**Objective:**

Whether or not reactivation of hepatitis B virus (HBV) might occur during corticosteroid therapy in hepatitis B surface antigen (HBsAg)‐negative patients with adrenal insufficiency was investigated.

**Patients and Methods:**

We consecutively enrolled 66 patients with adrenal insufficiency undergoing physiological corticosteroid replacement therapy at Saitama Medical University Hospital between June 2013 and June 2014, and 220 patients with rheumatic disease receiving a pharmacologic dose of corticosteroids served as the positive control group. The latter group was separated into 101 patients treated only with corticosteroids, and 119 patients given corticosteroids plus immunosuppressants and/or disease‐modifying antirheumatic drugs (DMARDs). HBsAg and antibody (Ab) levels against HBs, and hepatitis B core (HBc) were determined in all the patients. In patients with positive HBsAb and/or HBcAb, real‐time PCR was performed for HBV‐DNA. The incidence rates of conversion to HBV‐DNA‐positive status were evaluated.

**Results:**

Hepatitis B virus reactivation occurred in six patients with rheumatic disease, three of whom were receiving a pharmacological dose of corticosteroids only, and three who were receiving corticosteroids with immunosuppressants and/or DMARDs. However, no reactivation occurred in patients receiving corticosteroid replacements for adrenal insufficiency. Maintenance and maximum corticosteroid doses administered to patients with rheumatic disease were significantly greater than those in patients with adrenal insufficiency.

**Conclusion:**

These results suggest that, although corticosteroid replacement therapy for adrenal insufficiency might be safe with respect to HBV reactivation, attention should be paid to HBV reactivation during corticosteroid therapy in rheumatic disease patients, since the dose of corticosteroids administered is usually large, and since other immunosuppressants are co‐administered.

## 1 INTRODUCTION

Hepatitis B virus (HBV) reactivation occurring in patients undergoing cytotoxic chemotherapy and/or immunosuppressive therapy is a well‐recognized complication of considerable clinical importance. Reactivation of HBV is more often seen in hepatitis B carriers who are positive for hepatitis B surface (HBs) antigen (Ag) and for antibody (Ab) against hepatitis B core (HBc). Acute hepatitis, that is, de novo hepatitis, is likely to occur because of HBV reactivation during chemotherapy and/or immunosuppressive therapy given to patients with transplanted organs and leads to variable manifestations that range from subclinical serum aminotransferase elevation to fatal fulminant hepatitis. However, a few cases of HBV reactivation in patients with resolved infection as defined by negative HBsAg and positive HBcAb with or without HBsAb have been reported during chemotherapy or immunosuppressive therapy.^1^, ^2^ The incidence of de novo hepatitis due to HBV reactivation in HBsAg‐negative patients with malignant lymphoma treated with corticosteroid‐containing cytotoxic chemotherapy was reported to be 2.7% over a 4‐year period and 3.3% during a 12.4‐month follow‐up.^3^, ^4^ However, use of rituximab, an anti‐CD20‐directed monoclonal antibody, in combination with corticosteroid‐containing chemotherapy, has been frequently associated with HBV reactivation and resultant de novo hepatitis.^4^, ^5^, ^6^ Most of these reports come from the fields of oncology and transplantation, with a growing number of cases being reported in patients with rheumatic disease who are also undergoing immunosuppressive therapy. The incidence of HBV reactivation was reported to be 7/135 (5.2%) after a 1‐year prospective observation in rheumatoid arthritis patients with resolved hepatitis B treated with corticosteroids, immunosuppressants and/or disease‐modifying antirheumatic drugs (DMARDs).^7^ A recent prospective observational study in Japan has reported that HBV reactivation among rheumatic disease patients with resolved HBV infections treated with prednisone, immunosuppressants and/or biological DMARDs, occurred in 35/1042 (3.4%) of individuals over a period of 2 years.^8^ Estimated incidence rates of HBS reactivation and hence de novo hepatitis vary, likely due to differences among the patient populations studied, drugs administered and the duration of follow‐up.

Of all the traditional immunosuppressive medications, corticosteroids have been most often implicated in the induction of HBV reactivation. The risk of HBV reactivation in patients with inflammatory bowel disease, vasculitis, sarcoidosis and autoimmune disease might differ, depending on whether the patients are HBsAg‐positive/HBcAb‐positive or HBsAg‐negative/HBcAb‐positive, whether the dosage of chronic prednisone therapy is low, moderate or high, or whether the duration is short or longer than 4 weeks.^8^, ^9^ However, the exact incidence rate of HBV reactivation in HBsAg‐negative/HBcAb‐positive patients with autoimmune disease such as rheumatoid arthritis treated only with corticosteroids has not been fully evaluated.

On the other hand, adrenal insufficiency is usually treated with optimal replacement dose corticosteroids, equivalent to 5 mg of prednisone/day, to achieve physiological plasma cortisol levels comparable to those in healthy individuals. The incidence of HBV reactivation in patients with adrenal insufficiency given a physiological dose of corticosteroids is also unknown. Therefore, we investigated the incidence of the reactivation of HBV in HBsAg‐negative patients in adrenal insufficiency patients with corticosteroid therapy. And we also evaluated the incidence rate of HBV reactivation in patients with rheumatic disease treated with corticosteroids with or without immunosuppressants and/or DMARDs as the positive control.

## 2 PATIENTS AND METHODS

From June 2013 to June 2014, at Saitama Medical University Hospital, we consecutively enrolled and investigated 66 HBsAg‐negative patients with primary or secondary adrenal insufficiency who were undergoing corticosteroid replacement therapy. We also consecutively enrolled and evaluated 220 HBsAg‐negative patients with rheumatic disease who were receiving a pharmacological dose of corticosteroids as the positive control. Of these, 101 patients were treated solely with corticosteroids, while 119 patients were treated with both corticosteroids and immunosuppressants and/or DMARDs. HBsAb, HbcAb and HBsAg were measured at the beginning of the study. HBsAb and HbcAb were measured every three to 6 months and at the end of study. In patients who were positive for HBsAb and/or HBcAb, real‐time PCR was performed for HBV‐DNA. Daily maintenance and maximum doses of corticosteroids (prednisone or equivalent) were evaluated retrospectively from their medical charts. During the course of treatment, the dose of corticosteroids was decreased and held stable: this was regarded as the maintenance dose. The current study was approved by the Institutional Review Board of Saitama Medical University Hospital, and the informed consent was obtained from all participants.

The levels of HBsAg, HBsAb and HBcAb were determined in all the patients at the beginning of the study. An HBsAg level of <0.04 IU/mL was considered to be negative. Anti‐HBs levels of ≥10 IU/L were defined as positive, as were HBcAb levels ≥1.0 s/co. Real‐time PCR was performed on HBV‐DNA in patients who were positive for HBsAb and/or HBcAb, and the resulting HBV‐DNA level was considered to be positive if the level was ≥2.1 log copies/mL. Patients who were converted to HBV‐DNA‐positive status were defined as those who were HBsAg‐negative at the start of the treatment but became HBV‐DNA‐positive during treatment. We determined the incidence rates of conversion to HBV‐DNA‐positive status in patients with adrenal insufficiency or rheumatic disease. The levels of aspartate transaminase (AST), alanine transaminase (ALT), lactate dehydrogenase (LDH) and γ‐GTP were also measured during the observation period to assess liver function.

### 2.1 Statistical analysis

All the data are shown as mean ± SE. The chi‐squared test and ANOVA followed by a Tukey‐Kramer test were performed for comparisons, using JMP 9 statistical software. A *P*‐value of less than 0.05 was considered to be statistically significant.

## 3 RESULTS

A total of 66 patients (32 men and 34 women) had begun, or were receiving, corticosteroid replacement therapy for adrenal insufficiency. The diagnosis of diseases that result in adrenal insufficiency is shown in Table 1. The most common diagnosis was generalized hypopituitarism (n = 44), followed by isolated ACTH deficiency (n = 8), primary or postoperative adrenal insufficiency (n = 8), and so on. On the other hand, 220 patients (48 men and 172 women) were on corticosteroid treatment with or without immunosuppressants and/or DMARDs for various rheumatic diseases. Rheumatoid arthritis was the most common disease, with 80patients, followed by systemic lupus erythematosus (n = 46), overlap syndrome (n = 19) and polymyalgia rheumatica (n = 13), and so on.

**Table 1:** Diagnosis of the patients' underlying diseases

Group	Disease	No. of patients
Patients with adrenal insufficiency	Panhypopituitarism	44
	Isolated ACTH deficiency	8
	Sheehan's syndrome	2
	Lymphocytic adenohypophysis	2
	Primary hypoadrenocorticism	2
	Postoperative hypoadrenocorticism	6
	Congenital adrenal hyperplasia	2
Patients with rheumatic disease	Behçet's disease	4
	Mixed connective tissue disease	8
	Microscopic polyangiitis	5
	Polymyositis・Dermatomyositis	8
	Polymyalgia rheumatica	13
	Rheumatoid arthritis	80
	Systemic lupus erythematosus	46
	Adult Still's disease	5
	Systemic scleroderma	7
	Overlap syndrome	19
	Other collagen disease	25

Table 2 shows the patients' characteristics. Individuals with rheumatic disease were older than those with adrenal insufficiency. The duration of corticosteroid therapy did not differ among the three groups. The daily maintenance dose of corticosteroids was significantly different among the three groups (*P* < 0.0001). The doses of corticosteroids (prednisone or equivalent), that is 6.6 ± 0.5 mg and 6.5 ± 0.5 mg in patients with rheumatic disease treated with corticosteroids alone, and corticosteroids plus other immunosuppressants and DMARDs, respectively, were significantly larger than those (4.2 ± 0.2 mg) in patients with adrenal insufficiency (*P* < 0.05). Maximum daily doses of corticosteroids differed significantly among the three groups (*P* < 0.0047). The dose of corticosteroids in patients with rheumatic disease treated with only corticosteroids or corticosteroids plus other immunosuppressants and/or DMARDs, that is 24.1 ± 1.8 and 18.9 ± 1.8 mg, respectively, were significantly greater than 4.2 ± 0.2 mg in patients with adrenal insufficiency (*P* < 0.05). The levels of AST, ALT, LDH and γ‐GTP in the three groups are also shown in Table 2. No statistically significant difference was noted in these liver function tests.

**Table 2:** Characteristics of the study population

	Patients with adrenal insufficiency	Patients with rheumatic disease	
		Steroids only	Steroids + biological drugs
No. of patients	66	101	119
Sex M/F	M 32 F 34	M 20 F 81	M 28 F 91
Age (years)	53.5 ± 2.2 (19‐88)	60.3 ± 1.6 (19‐84)	61.0 ± 1.5 (19‐94)
Duration of therapy (years)	8.8 ± 1.0 (1‐42)	10.2 ± 0.9 (1‐40)	9.1 ± 0.7 (1‐35)
Maintenance dose (mg)	4.2 ± 0.2 (0.625‐10)	6.6 ± 0.5 (1‐20)	6.5 ± 0.5 (0.5‐40)
Maximum dose (mg)	4.2 ± 0.2 (0.625‐10)	24.1 ± 1.8 (3‐60)	18.9 ± 1.8 (1‐80)
AST (μ/L)	26.4 ± 1.1	23.1 ± 1.0	23.7 ± 1.1
ALT (μ/L)	27.0 ± 2.2	20.0 ± 1.5	19.1 ± 0.9
LDH (μ/L)	190.1 ± 3.9	212.8 ± 4.8	215.8 ± 4.2
γ‐GTP (μ/L)	30.9 ± 3.9	35.8 ± 8.0	36.2 ± 4.4

Note

Data are mean ± SE (range).

Abbreviations: ALT, alanine transaminase; AST, aspartate transaminase; LDH, lactate dehydrogenase; γ‐GTP, γ‐glutamyltranspeptidase.

As shown in Table 3, the number (percentage) of patients who showed negative for both HBsAb and HBcAb was 55/66 (83.3%), 89/101 (85.1%) and 100/119 (84.0%) in the adrenal insufficiency group, the rheumatic disease group treated with only corticosteroids, and the rheumatic disease group treated with immunosuppressive drugs and/or DMARDs, respectively. On the other hand, 11 patients showed positive for HBsAb and/or HBcAb in the adrenal insufficiency group, compared with 15 patients in the rheumatic disease group treated only with corticosteroids and 19 patients treated with immunosuppressive drugs and/or DMARDs, with no significant difference among the groups.

**Table 3:** Results of HBsAb and HBcAb measurements

	Patients with adrenal insufficiency	Patients with rheumatic disease	
		Steroids only	Steroids + biological drugs
No. of patients	66	101	119
HBsAb (−)・HBcAb (−)	55	86	100
HBsAb (−)・HBcAb (+)	0	3	4
HBsAb (+)・HBcAb (−)	3	1	3
HBsAb (+)・HBcAb (+)	8	11	12

Abbreviations: HBcAb, antibody against hepatitis B core; HBsAb, antibody against hepatitis B surface; HBsAg, hepatitis B surface antigen.

As shown in Table 4, among those with resolved HBV infections, three patients each (2.9% and 2.5%) had converted to HBV‐DNA‐positive status in the rheumatic disease group treated only with corticosteroids, and with corticosteroids plus immunosuppressive drugs and/or DMARDs, but none had converted in the adrenal insufficiency group, although the difference among the three groups did not reach a significant level. The six patients who had converted to HBV‐DNA‐positive status were aged between 46 and 78 years; of these, five were aged 60 years and older. Five of the six patients were female. The duration of corticosteroid therapy in these patients ranged from 1 to 30 years. The maintenance daily dose of corticosteroids ranged from 2 to 45 mg. The maximum daily dose of corticosteroids ranged from 5 to 60 mg. Furthermore, all the patients were positive for HBcAb, and three were positive for HBsAb (Table 4). These six patients did not present any symptoms or signs of hepatitis, such as generalized fatigue or jaundice. None of the patients developed de novo hepatitis or fulminant hepatitis.

**Table 4:** Characteristics of HBV‐reactivated patients

Case	Age (years) and sex (M/F)	HBsAb	HBcAb	HBV‐DNA (Log copies/mL)	ALT (U/L)	Maintenance dose of corticosteroids (mg)	Maximum dose of corticosteroids (mg)	Therapy duration (years)	Concomitant medication
1	68 F	+	+	3.4	10	5	5	30	None
2	46 F	−	+	2.1	11	3	60	30	None
3	62 M	−	+	2.1	16	8	60	22	None
4	71 F	+	+	2.1	44	2	5	13	Tacrolimus hydrate, methotrexate
5	78 F	+	+	2.1	11	45	45	1	Melphalan
6	75 F	−	+	3.7	7	6	6	4	Abatacept, methotrexate

Abbreviations: ALT, alanine transaminase; HBcAb, antibody against hepatitis B core; HBsAb, antibody against hepatitis B surface; HBsAg, hepatitis B surface antigen; HBV‐DNA, hepatitis B virus‐DNA.

It should be noted that, among the patients who had converted to HBV‐DNA‐positive status, corticosteroids were administered as monotherapy in three patients and as combination therapy in three patients. Of these patients, one was co‐administered with melpharan, while two were co‐administered with methotrexate and either tacrolimus or abatacept (Table 4).

## 4 DISCUSSION

In this study, we investigated the incidence of HBV reactivation among HBsAg‐negative patients treated with a physiological dose of corticosteroids as replacement therapy and a pharmacological dose for anti‐inflammatory and/or immunosuppressive purposes. First of all, patients who showed negative for both HBsAb and HBcAb at the beginning of and/or during the study corresponded to about 85% of the patients included in the study. They were believed to have not been exposed to HBV, although we cannot completely rule out the possibility that several patients had been exposed to HBV but turned out to be seronegative, that is, HBsAg‐negative. Those patients who had never been exposed to HBV during the study period provide no value in a study to elucidate the effects of corticosteroids on the reactivation of HBV. Still, it is meaningful to learn about how many patients were exposed to HBV, and how many were not during the study period. In the present study, 11 patients showed positive for HBsAb and/or HBcAb in the adrenal insufficiency group, compared with 15 patients in the rheumatic disease group treated with only corticosteroids, and 19 patients treated with immunosuppressive drugs and/or DMARDs. Generally speaking, positive HBcAb suggests previous HBV infection resulting in natural immunity, while positive HBsAb and negative HBcAb suggest previous successful vaccination resulting in immunity, rather than infection with HBV. However, in Japan, vaccination against HBV has been recommended only to infants born from HBsAg‐positive mothers since 1986. HBV vaccination has been also recommended to subjects who often come into contact with blood and other body fluids, such as medical and police personnel, but not widely accepted. Later, beginning 1 October 2016, all newborns were strongly advised to undergo HBV vaccination as part of the routine immunization program by the age of one. Practically, all newborns are now vaccinated against HBV. We have confirmed that three patients in the adrenal insufficiency group and four patients in the rheumatic group with positive HBsAb and negative HBcAb did not receive HBV vaccinations. The discrepancy between positive HBsAb and negative HbcAb and vice versa might be attributable to the period during which HBsAb and HBcAb were measured, since, when infection with HBV occurs, HBcAb usually turns positive immediately after exposure to HBV, and, while its potency may or may not weaken, HBsAb antibodies subsequently turn positive. In any event, fortunately, none had HBV reactivation in the adrenal insufficiency group, although, in the latter group consisting of 220 patients with rheumatic disease, HBV reactivation occurred in six cases, that is, 2.7%. We found no significant differences in the results of liver function tests between recurrent and nonrecurrent patients. This is consistent with the previous study which showed no differences in serum liver enzyme levels between patients with and without recurrent hepatitis B.^5^, ^7^ Fortunately, none of the patients developed de novo hepatitis in this study.

Secondarily, our results in patients with rheumatic disease confirmed the previous studies in patients with rheumatic arthritis and other rheumatic diseases with resolved HBV infection conducted in Japan, which have reported that HBV reactivation occurred after administration of immunosuppressive agents and DMARDs at the rate of 5.2% during a 1‐year period or 3.4% during a 2‐year follow‐up,^7^, ^8^ respectively. However, the use of rituximab in combination with corticosteroids has been recently shown to increase the risk of HBV reactivation.^4^, ^5^, ^6^, ^10^ On the other hand, three out of 101 patients with rheumatic disease (2.9%) treated with corticosteroids only in the present study experienced HBV reactivation, confirming that of the traditional immunosuppressive medications, corticosteroids have most often been implicated as a significant predisposing factor for HBV reactivation. In fact, rheumatic disease patients treated with prednisone showed a higher risk ratio of 2.2 (95% confidence interval 1.0 to 4.6, *P* < 0.04) than those treated without prednisone.^8^

Resolved HBV infection is defined as an HBsAg‐ and HBV‐DNA‐negative status, but anti‐HBcAb or anti‐HBsAb remains positive. HBV exists as covalently closed circular DNA (cccDNA) in hepatocytes and mononuclear cells, even in patients with resolved HBV infection.^9^ There are several possible mechanisms for corticosteroid‐induced HBV reactivation. The first mechanism might be that corticosteroids directly cause HBV to proliferate because HBV has a glucocorticoid‐responsive element that has the same base sequence as the glucocorticoid receptor (GR), a hormone‐dependent nuclear receptor, at the initiation site of the viral gene.^11^ The second mechanism involved may be an indirect immunosuppressive effect on the host immune response which facilitates HBV replication. HBV persists for decades after patients' recovery from acute viral hepatitis, despite active maintenance of a cytotoxic T‐lymphocyte response.^12^ Glucocorticoid inhibits induction of gene expression of inflammatory cytokines and chemokines via action on NF‐κB and activation protein (AP)‐1. One study has reported that prednisone administered at a dose of 30 mg/d is sufficient to induce the genomic effects of corticosteroids in the body.^13^ In fact, a corticosteroid dose equivalent to 30 mg/d of prednisone results in the proliferation of HBV and imposes a risk of HBV reactivation associated with high‐dose steroids.

In contrast, it should be noted that we had no cases of HBV reactivation among adrenal‐insufficient patients on corticosteroid replacement therapy. The daily maintenance and maximum doses of corticosteroids were both 4.2 ± 0.2 mg, which were significantly lower than those prescribed to rheumatic disease patients. In recent reviews by Perrillo et al^14^ and Loomba and Liang,^15^ HBsAg‐positive/HBc antibody‐positive patients with inflammatory bowel disease, vasculitis, sarcoidosis and autoimmune disease who were treated with corticosteroids over 4 weeks at moderate (10‐20 mg/d prednisone or equivalent) or high doses (more than 20 mg/d prednisone or equivalent), were reported to be at a moderate or high risk of reactivation of HBV, the incidence of which was 1%‐10% or more than 10% of cases. HBsAg‐positive/HBcAb‐positive patients receiving a low dose of corticosteroids of <10 mg/d prednisone or equivalent over 4 weeks are regarded as being at low risk, and their incidence of HBV reactivation is estimated to be <1.0%. On the other hand, HBsAg‐negative/HBcAb‐positive patients with inflammatory and autoimmune disease treated with a high dose of corticosteroids of more than 20 mg/d prednisone or equivalent over 4 weeks, are regarded to be at moderate risk, and those treated with moderate or low dose of corticosteroids at 10‐20 mg/d or <10 mg/d prednisone or equivalent over 4 weeks, are regarded to be at low risk with an incidence rate of <1.0%. Based on the current study, the incidence of HBV reactivation in rheumatic disease patients treated at low doses of corticosteroids (6.5 mg prednisone or equivalent) might be lower than those reported in these reviews. Furthermore, no HBV reactivation occurred in patients with adrenal insufficiency whose treatment was replaced with a physiological dose of corticosteroids, that is, 4.2 mg of prednisone or equivalent per day, indicating that replacement therapy might be safe with respect to HBV reactivation. However, it should be noted that, in an inactive HBV carrier, HBV reactivation had occurred during a very low‐dose steroid treatment (2.5 mg of prednisone per day plus DMARDs such as sulfasalazine and hydroxychloroquine).^16^

It has been reported that a greater number of HBcAb‐positive and HBsAb‐negative patients experience HBV reactivation, compared with patients who are HBcAb‐negative and anti‐HBs‐positive.^6^, ^17^ In our study, all six HBV‐positive patients were positive for HBcAb, suggesting that HBcAb has considerable potential as a marker and screening tool for patients with resolved HBV infection. Our results suggest that special attention should be given to the possible reactivation of HBV in patients who are positive for HBcAb.

In our study, patients who experienced HBV reactivation were aged between 46 and 68 years, with five of the six patients aged over 60. Other studies have reported age to be significantly associated with HBV reactivation in HBsAg‐negative patients with B‐cell lymphoma or rheumatic disease treated with rituximab plus corticosteroid‐containing chemotherapy or corticosteroids, immunosuppressive drugs and/or DMARDs.^8^, ^10^ However, some rheumatic disease patients in their 20s and 30s treated with immunosuppressive and biological drugs were reported to show HBV reactivation.^18^ Moreover, in one Japanese study that evaluated 135 resolved hepatitis B patients with rheumatoid arthritis, age was not a predictive factor for HBV reactivation.^7^ Therefore, attention should be paid to not only the elderly but also to younger individuals with respect to HBV reactivation, especially when biological DMARDs are being co‐administered with corticosteroids. In addition, although the male gender has been reported to be more associated with HBV reactivation in patients with chemotherapy‐treated lymphoma,^3^, ^5^ five of the six patients in our present study were female. This is consistent with previous studies in rheumatic disease which report that the male gender was not necessarily associated with HBV reactivation on corticosteroids with or without biological agents.^7^, ^8^

Hui et al 4 reported temporal changes in HBV reactivation following chemotherapy in patients with resolved hepatitis B infection. It has been reported that the median time to convert to positive HBV‐DNA was 12 weeks, the median time from HBV‐DNA positivity to reappearance of HBsAg was 10 weeks, and the median time from reappearance of HBsAg to the onset of acute hepatitis was 18.5 weeks.^4^ Another report has described the occurrence of hepatitis after an average of 49 days following chemotherapy.^19^ In our study, however, the duration of corticosteroid therapy varied from 1 to 30 years in six rheumatic disease patients who had converted to HBV‐DNA‐positive status, suggesting that conversion to positive HBV‐DNA might be independent of treatment duration. Periodic assessment of HBV‐DNA levels as well as HBsAg may be important during corticosteroid therapy with or without immunosuppressive and molecular‐targeted drugs.

This study has several limitations. First, it was designed as a cross‐sectional observational study, not a randomly controlled prospective study. Second, the rate of incidence of HBV reactivation did not show a statistically significant difference among the three groups. Nevertheless, the difference is clear at a glance: HBV reactivation occurred in none of the 66 patients with adrenal insufficiency who were treated with a physiological dose of corticosteroids, but did occur in three out of 101 patients with rheumatic disease given a pharmacological dose of corticosteroids only, and in three out of 119 patients with rheumatic disease given a pharmacological dose of corticosteroids, together with immunosuppressive drugs and/or DMARDs. This might be due to the small number of patients observed and the relatively short observation period of only 13 months. To guarantee the safety of replacement therapy with a physiological dose of corticosteroids in patients with adrenal insufficiency, and to dispel their concerns for HBV reactivation, further prospective studies will be needed, with a longer observational period and with a larger population of patients with adrenal insufficiency. This should also apply to patients with rheumatic disease, to further ascertain the influence of treatment comprising corticosteroids and immunosuppressants with or without DMARDs.

In conclusion, our present study suggests that, although corticosteroid replacement therapy for adrenal insufficiency might be safe with respect to HBV reactivation, we should remain alert to the risk of HBV reactivation during corticosteroid therapy in patients with rheumatic disease, since the dose of corticosteroid administered is usually high, especially at the beginning, and since other immunosuppressive and molecular‐targeted drugs are co‐administered.

## CONFLICT OF INTEREST

Nothing to declare.

## AUTHOR CONTRIBUTION

S Katayama, T Mimura, A Shimada and M Noda conceptualized and designed the study. M Hatano contributed in ethics submission, review of literature, data collection, analysis, interpretation of results and elaboration of manuscript and submission. S Katayama and M Hatano wrote the manuscript and T Mimura, A Shimada and M Noda substantially revised the manuscript.

## ETHICAL STATEMENT

This study was approved by the Institutional Review Board of Saitama Medical University Hospital (No. 15‐053‐1) and the informed consent was obtained from all participants.

## Data Availability

All data generated or analysed during this study are included in this published article.
