# Corrigendum

**DOI:** 10.1002/edm2.138

**Published:** 2020-04-20

In the article by Hatano et al.,^1^ the authors wish to acknowledge that three cases of their control group were kept on immunosuppressive therapy since 2011; thus there is an overlap of a small period of time (a year and 3 months: from the start of 2011 until 31 March 2012) with the Part 2 study of Mochida et al.^2^ This fact was inadvertently omitted in the published article. The authors have reanalysed the data by omitting these three cases and confirm that the conclusions of their study are unchanged.

The authors apologize for this omission from their published article.
